# Determinants of Quality of Life Among Hemodialysis Patients: Roles of Associated Comorbidities, Smoking, Climate Change, and Residence

**DOI:** 10.1155/jnme/6370912

**Published:** 2026-07-01

**Authors:** Amany Gomaa Atia, Hani Jameel Hamad, Mohamed Ali Elsayed, Huda Badr Abd Elhamed, Hanaa Elsayed Elsayed Mohamed, Shimaa Galal El Sayed

**Affiliations:** ^1^ Department of Physical Therapy for Surgery, Faculty of Physical Therapy, Sinai University, Ismailia, Egypt, su.edu.eg; ^2^ Department of Clinical Nutrition and Dietetics, Faculty of Allied Medical Sciences, Philadelphia University, Amman, 19392, Jordan, philau.edu; ^3^ Department of Physical Therapy, Faculty of Allied Medical Sciences, Philadelphia University, Amman, Jordan, philau.edu; ^4^ Department of Musculoskeletal Disorders and Its Surgery, Faculty of Physical Therapy, Cairo University, Giza, Egypt, cu.edu.eg; ^5^ Department of Physical Therapy for Internal Medicine and Geriatrics, Innovation University, sharkia, Egypt; ^6^ Department of Physical Therapy for Surgery, Faculty of Physical Therapy, Ahram Canadian University, Giza, Egypt, acu.edu.eg

**Keywords:** climate change, Egypt, hemodialysis, quality of life, smoking

## Abstract

**Problem considered:**

Hemodialysis patients with end‐stage renal disease (ESRD) frequently experience impaired health‐related quality of life (HRQoL) due to multifactorial influences, including comorbidities, environmental stressors, and lifestyle factors. Hence, this study aimed at evaluating the determinants of HRQoL of hemodialysis patients, with a focus on the roles of comorbidities, cigarette smoking, environmental exposure, and residential setting.

**Methods:**

A cross‐sectional observational study of 84 adult (ranging in age from 18 to 65 years) patients undergoing maintenance hemodialysis was conducted at Hehia Hospital in Egypt for one year, extending from May 2024 to May 2025. The study sample consisted predominantly of middle‐aged to older adults (mean age 55.33 ± 12 years), with a majority being male (64.3%), having primary education or less (46.4%), and residing in rural areas (83.3%). The HRQoL was assessed using the Kidney Disease Quality of Life Short Form (KDQOL‐SF). Sociodemographic, clinical, and environmental data were collected. Univariate and multivariate regression analyses were conducted to identify significant predictors of the overall QoL of hemodialysis patients with ESRD.

**Results:**

Multivariate analysis revealed that educational attainment (*β* = −0.295, *p* = 0.002), presence of comorbid conditions (*β* = 0.178, *p* = 0.043), occupational exposure to excessive climate (*β* = −0.183, *p* = 0.039), smoking status (*β* = 0.204, *p* = 0.021), perceived stress (*β* = 0.180, *p* = 0.039), and pain intensity (*β* = 0.322, *p* = 0.001) were significant predictors of the HRQoL of the hemodialysis patients with ESRD. These predictors, together, explained 45.5% of the variance in the QoL scores of the patients (*R* ^2^ = 0.455). Notably, smokers had significantly lower HRQoL scores than the nonsmokers.

**Conclusion:**

The HRQoL of hemodialysis patients is adversely affected by smoking, pain, stress, educational background, comorbid diseases, and climate exposure. These findings underscore the need for holistic, patient‐centered interventions that incorporate smoking cessation, psychosocial support, and environmental adaptations to improve the well‐being of this vulnerable population.

## 1. Introduction

Among the therapies available for end‐stage renal disease (ESRD) are conservative care and renal replacement therapy (RRT), both of which can lower the health‐related quality of life (HRQoL) and increase morbidity and mortality [1, 2]. Improving the patient’s well‐being in addition to their survival is one of the objectives of RRT for ESRD patients [3]. Each of the various RRT modalities, such as kidney transplantation (KT), hemodialysis (HD), and peritoneal dialysis (PD), has unique benefits and drawbacks [4]. HD is the most common dialysis therapy type worldwide, accounting for nearly 80% of global use, followed by PD and KT [1, 5, 6]. Previous research has shown that the HRQoL of dialysis patients is poorer than that of the community as a whole [1, 4].

According to recent studies, dialysis patients consistently have much lower HRQoL than the general population. The mean HRQoL score of dialysis‐dependent patients in India was 32.70 ± 6.00, which is significantly lower than population norms [7]. In a similar vein, a multicenter study conducted in Albania found that HD patients had mean Physical Component Summary scores of 34.17 ± 12.99 and Mental Component Summary scores of 47.52 ± 13.95, with multiple comorbidities, female gender, and older age being the significant contributing factors [8]. Furthermore, even though social support networks can lessen some of the burden, a study conducted in Lebanon and Canada revealed that HD patients have lower HRQoL than the general population [9]. Overall, these results highlight the significant effects of dialysis on mental health, physical functioning, and general quality of life (QoL).

The concept of HRQoL is a multifaceted concept that encompasses mental, emotional, behavioral, and physical aspects of functioning. It has a tight connection to the “well‐being” concept. It quantifies the impact of health on (QoL) by going beyond simple indicators of health like mortality and life expectancy [10, 11]. The HRQoL has been assessed using a variety of instruments that differ, primarily, in the characteristics that are measured and the level of precision with which they are measured [12, 13]. The HRQoL is a valuable means of assessing the decline in a smoker’s QoL before the onset of chronic illnesses and early mortality, which is a component of the economic burden of smoking that is frequently disregarded [14, 15]. Dialysis interferes with the lives of the patients and their families and alters their way of living greatly. Career, eating patterns, daily tasks, self‐worth, safety, community interactions, and the ability to take pleasure in life are among the main aspects affected by ESRD and its therapy [16, 17]. Long‐term HD patients frequently worry about the uncertainty of their health condition and the disruption of their lives that it may cause. They often struggle financially, have trouble keeping a job, experience impotence and waning sexual drive, suffer from depression because of this chronic illness, and fear death [16, 18]. Younger patients are primarily concerned about getting married, establishing a new family, and managing the associated financial strain [17]. Frequent dialysis treatments and dietary and fluid restrictions impose a structured lifestyle that can be discouraging, both for the patients and for their families [16]. Prior studies comparing men and women have shown that QoL varies according to gender‐related disadvantages, e.g., women’s poorer socioeconomic status and higher widowhood rates [19, 20]. There is an ongoing debate on the significance of gender in QoL analysis. Research on gender variations in QoL in Finland, Japan, and Israel has repeatedly shown that women generally have lower QoL than men, even though they have an established survival advantage over men almost everywhere in the world [19, 20].

The current investigations indicate that hypertension may contribute to a decline in the HRQoL of the patients [21, 22]. The chronic and deleterious nature of this condition; the diagnosis itself; its detrimental effects on social, emotional, and physical functioning; and the effects of medication therapy are some of the main factors that impact the QoL of people with hypertension [23, 24]. According to recent research, the QoL of individuals with Type 2 diabetes is lowered considerably by consequences such as myocardial infarction, neuropathy, retinopathy, and hospitalization for unstable angina pectoris [25].

Furthermore, new research demonstrated that climate change has a substantial negative impact on wellness, community, and financial aspects of QoL. Exposure to extreme heat is associated with higher levels of anxiety and depression, which have a direct impact on mental health [26]. Financially vulnerable households cannot afford to cool their homes, which exacerbates social and economic inequality and forces them to make difficult decisions between their health and financial stability [27]. Rising urban temperatures are also linked to emotional distress and challenges with everyday tasks, underscoring climate change as a global systemic threat to well‐being [28].

In consequence, a comprehensive strategy that incorporates scientific understanding, legislative actions, and community‐based solutions is necessary to address the various effects of climate change on living standards. From increased susceptibility to heat‐related disorders and the proliferation of vector‐borne diseases, environmental degradation has both direct and indirect implications on well‐being [29]. Health problems can be made worse by rising temperatures and shifting precipitation patterns, particularly for vulnerable groups with little access to medical treatment. Hurricanes and wildfires are examples of extreme weather phenomena that pose immediate risks to life and well‐being. They result in harm, relocation, and persistent illness effects [29].

Recent research shows that work‐related thermal stress has a significant negative impact on kidney health. Zhang et al. [30] found that in more than 4000 patients with chronic kidney disease, each 30‐day high temperature exposure (heat index > 30 degrees Celsius) was associated with a −0.6% decrease in eGFR, resulting in an additional 3.7 mL min and 1.73 m^2^ annual loss in hot climates. A study by Amorim and Schlader [31] found that hyperthermia causes tissue hypoxia and subclinical renal insufficiency through reducing renal blood flow and increasing metabolic demand. Importantly, a study by Hansson et al. [32] conducted over four harvests reported that low‐cost workplace interventions such as rest, shade, hydration, and hygiene reduced the incidence of acute renal injury from 21 to only 1% among sugarcane workers. This is an indication of the effectiveness and economic viability of conservation measures.

A review of the literature revealed few studies that examined the connections between HD patients’ HRQoL and cigarette smoking. Although smoking has been shown to have detrimental effects on mortality and cardiovascular outcomes in this population [33], its relationship to HRQoL is still poorly understood. A study by Østhus et al. [34] showed that smoking is independently linked to lower mental HRQoL and depression, whereas Molsted et al. [35] revealed that low HRQoL scores are predicted by smoking rather than by conventional quality parameters. There are, however, contradictory results; Emiroğlu et al. [36] revealed that smoking was linked to improved physical HRQoL in younger patients, showing possible confounding variables or reverse causality that call for more research.

Moreover, Tohme et al. [37] found a reciprocal relationship in which treatment nonadherence is predicted by both smoking and low HRQoL, indicating a complicated interaction that is still unclear. More investigation is required to determine the type and extent of the relationship between smoking and HRQoL in HD patients in light of these contradictory results and the scant amount of data. Therefore, the purpose of this study was to examine the associations between HD patients’ QoL and potential determinants such as associated comorbidities, smoking status, climate change factors, and place of residence.

## 2. Methods

### 2.1. Study Design and Setting

This cross‐sectional observational study was carried out in the nephrology unit of Hehia Hospital, a public tertiary care facility in Egypt, during a year extending from May 2024 to May 2025. This hospital offers comprehensive renal care services, including scheduled outpatient HD. G∗Power software (Version 3.1.9.4) was employed to calculate the sample size for this study so as to guarantee sufficient statistical power. A sample size of 76 subjects was recommended by this software. It provides 80% power on the basis of a two‐tailed correlation test at the level of statistical significance of 0.05, based on an expected modest association (*r* = 0.31) between smoking, associated disease, and QoL as reported in previous research. Over the course of the study, eighty‐five eligible patients were contacted. Eighty‐four of them provided complete data, rendering a sample size of 84.

### 2.2. Inclusion and Exclusion Criteria

The inclusion criteria were patients (i) ranging in age from 18 to 65 years, (ii) receiving maintenance HD, and (iii) who are able to understand Arabic, the formal language of the country. The participants were those patients who agreed to participate in this study and who filled and submitted the “*Informed written Consent*” form that was accepted by the Faculty of Physical Therapy at Cairo University. An unbiased literate witness obtained written informed consent for participants who were illiterate by watching the oral explanation of the study, confirming the participant’s comprehension and willingness to participate voluntarily, and cosigning the consent form with the researcher after the participant put their thumbprint on it. On the other hand, the exclusion criteria were (i) patients with any mental, communication, or hearing disorders that may make it difficult for them to administer the assessment; (ii) patients with continued mental health treatment; and (iii) patients who did not agree to participate in this study.

### 2.3. Participant Characteristics

Of the 84 participants, sixty‐eight patients were nonsmokers and 16 were smokers. Thirty‐eight of the patients were having hypertension, while 17 were suffering from cardiovascular diseases and 17 others were suffering from arthritis. Meanwhile, seven of the patients were diabetic patients and five patients were undergoing chemotherapy. In other respects, fourteen patients were living in urban areas, while the remainder 70 patients were living in rural areas.

### 2.4. Tools and Techniques

For data collection, this study employed the Kidney Disease Quality of Life Short Form (KDQOL‐SF). This instrument has 36 items that capture the participant’s assessment of her/his health over the preceding four weeks. These items address varying parameters, including symptoms and issues, the impacts of kidney disease, consequences of renal illness in day‐to‐day living, situation at work and mental abilities, quality of social interactions, sexual health, rest patterns, social assistance, assistance from dialysis staff, patient contentment, involvement in physical activities, pain levels, physical capacity, overall health, emotional roles, social engagement, and energy/fatigue levels. The Numeric Rating Scale, in regard to the pain scoring, is as follows: 0 = No Pain; 1–3 = Mild Pain (nagging, annoying, interfering little with Activities of Daily Living (ADLs)); 4–6 = Moderate Pain (interferes significantly with ADLs); and 7–10 = Severe Pain (disabling, unable to perform ADLs) [38]. Furthermore, patient’s demographic information, including gender, income, education, and daily drug use, is addressed by this instrument. After numerical precoding of all item responses, higher values indicate better health status. Raw scores are then linearly transformed to a standardized scale ranging from 0 (lowest possible health) to 100 (highest possible health) [39]. To ensure clarity of the instrument items and questions and resolve any problems in item comprehension, the instrument was first piloted on 5% of the target population before final implementation. Adaptations were made in response to participant input.

### 2.5. Data Analysis

This investigation was structured according to the guidelines of Strengthening the Reporting of Observational Studies in Epidemiology (STROBE) in order to guarantee methodological clarity and reproducibility. Data assessment was performed using the IBM SPSS software (v. 27), with the level of statistical significance (*α*) set at 0.05. Descriptive statistics are documented in this paper as means and standard deviations for normally distributed variables and as medians with interquartile ranges for non‐normally distributed variables (e.g., the SF‐36 physical and mental health domains). Results pertaining to categorical data are presented as frequencies and percentages. The incidence of perceived stress was evaluated via the Clopper–Pearson method for binomial proportions to establish 95% confidence intervals. Correlation analysis was performed using Pearson’s correlation analysis for normally distributed variables and Spearman’s rank correlation analysis for non‐normally distributed variables. Using univariate regression analysis, the dependent variable (overall QoL) was regressed on each independent variable (age, mass, height, body mass index (BMI), gender, marital status, residence, associated diseases, climate at work, smoking, education level, duration of dialysis, pain, and stress) separately to identify the significant (*p* < 0.05) predictors. Finally, all the independent variables which univariate analyses revealed to be significant predictors of the overall QoL of the HD patients with ESRD were employed as the predictors in a multivariate regression model. The SPSS package (SPSS Inc., Chicago, IL, USA) was the software used for all statistical analyses and modeling.

## 3. Results

The study sample consisted of 84 HD patients of both genders. The distribution of those patients according to their sociodemographic characteristics is illustrated in Table 1, while information on comorbidity, environmental stressors, and lifestyle factors are summarized in Table 2.

**TABLE 1:** Distribution of the study participants according to their sociodemographic characteristics.

Variable	*n*	Percentage (%)
Age (years); mean ± SD	55.33 ± 12	
Gender		
Male	54	64.3
Female	30	35.7
Marital status		
Married	56	66.7
Single	4	4.80
Widowed	20	23.8
Divorced	4	4.80
Education		
Primary	39	46.4
Preparatory	11	13.1
Secondary	21	25.0
Bachelor	13	15.5
Residence		
Urban	14	16.7
Rural	70	83.3

*Note: n*: number of participants.

Abbreviations: QoL = quality of life, SD = standard deviation.

**TABLE 2:** Baseline characteristics of the study participants (*n* = 84).

Variable	*n*	Percentage (%)
Mass (kg); mean ± SD	78.5 ± 16.0	
Height (cm); mean ± SD	162 ± 7.0	
BMI (kg/m^2)^; mean ± SD	29.8 ± 5.25	
Associated diseases		
HTN	38	45.2
CVDs	17	20.2
Arthritis	17	20.2
Chemotherapy	5	6
Diabetic	7	8.3
Duration of dialysis		
0–1 year	17	20.2
1–3 years	18	21.4
3–5 years	12	14.3
> 5 years	37	44
Smoking		
Smoker	16	19
Nonsmoker	68	81
Climate at work		
Exposed	30	35.7
Not exposed	54	64.3
Stress; mean ± SD	16.6 ± 4.6	
QoL‐total; mean ± SD	66.9 ± 21.5	
Pain (average); mean ± SD	5.31 ± 1.35	

*Note: n*: number of participants; kg: kilogram; cm: centimeter; m: meter; HTN: hypertension; CVDs: cardiovascular diseases.

Abbreviations: BMI = body mass index, QoL = quality of life, SD = standard deviation.

### 3.1. Univariate Analysis

In the current study, the dependent variable (overall QoL) was regressed on each independent variable (age, mass, height, BMI, gender, marital status, residence, climate at work, smoking, education level, associated diseases, duration of dialysis, pain, and stress) separately using univariate regression analysis to identify the significant (*p* < 0.05) predictors. The outcomes of this analysis (Table 3) show that age, education level, marital status, climate at work, smoking, stress, and pain are significant individual predictors of the overall QoL of the HD patients with ESRD.

**TABLE 3:** Outcomes of univariate regression analysis of predictors of the overall QoL of hemodialysis patients with ESRD.

Model	*B*	SE	Beta	*t*	Sig.	95% CI for *B*	
						Lower	Upper
Age	0.486	0.194	0.270	2.510	**0.01**	0.10	0.87
Gender	0.322	4.925	0.007	0.065	0.95	−9.48	10.12
Mass	0.069	0.150	0.051	0.458	0.65	−0.23	0.37
Height	−0.172	0.342	−0.056	−0.502	0.62	−0.85	0.51
BMI	0.379	0.454	0.092	0.835	0.41	−0.52	1.28
Education level	−7.597	1.871	−0.411	−4.061	**0.001**	−11.32	−3.88
Marital status	4.873	2.404	0.220	2.027	**0.05**	0.09	9.68
Residence	−6.542	6.498	−0.111	−1.007	0.32	−19.470	6.39
Duration of dialysis	0.028	1.972	0.002	0.014	0.99	−3.894	3.95
Associated diseases	2.540	1.043	0.260	2.435	**0.02**	0.465	4.62
Climate at work	−9.691	4.866	−0.216	−1.991	**0.05**	−19.373	−0.01
Smoking	11.800	6.035	0.212	1.955	**0.05**	−0.21	23.81
Stress	1.180	0.495	0.255	2.386	**0.02**	0.20	2.17
Pain	8.138	1.504	0.513	5.410	**0.001**	5.15	11.13

*Note:* Effect is significant if *p* < 0.05. The bolded values in the table represent statistically significant findings with a significance level set at *p* < 0.05.

Abbreviations: BMI = body mass index, CI = confidence interval, SE = standard error.

### 3.2. Multivariate Regression Analysis

All the predictors of the overall QoL of the HD patients with ESRD, which simple linear regression analysis proved to be significant (age, education level, marital status, climate at work, smoking, stress, and pain), were input as predictors to a multivariate regression model. The results of this analysis (Table 4) disclose that age and marital status are not significant predictors. Therefore, they were removed from the model as neither altered the effect (R‐squared) by more than 10%. Table 4 shows the results of multivariate regression analysis after these two variables had been excluded.

**TABLE 4:** Multivariate regression analysis of predictors of the overall QoL of hemodialysis patients with ESRD.

Model	*B*	SE	Beta	*t*	Sig.	95% CI for *B*	
						Lower	Upper
Constant	32.858	11.857		2.771	**0.01**	9.24	56.47
Education level	−5.456	1.669	−0.295	−3.268	**0.001**	−8.78	−2.13
Associated diseases	1.732	0.841	0.178	2.061	**0.04**	0.06	3.41
Climate at work	−8.229	3.912	−0.183	−2.104	**0.04**	−16.02	−0.44
Smoking	11.332	4.824	0.204	2.349	**0.02**	1.72	20.94
Stress	0.895	0.426	0.180	2.101	**0.04**	0.05	1.74
Pain	5.111	1.467	0.322	3.484	**0.001**	2.19	8.03

*Note:* Effect is significant if *p* < 0.05. The bolded values in the table represent statistically significant findings with a significance level set at *p* < 0.05.

Abbreviations: CI = confidence interval, SE = standard error.

The equation of the multivariate regression model can be written as follows:

Overall QoL = 32.86–5.46 × education level +1.73 × associated diseases −8.23 × climate at work + 11.33 × smoking + 0.9 × stress + 5.1 × pain.

This suggests that, for example, when the pain score increases from 1 to 2 (assuming constancy of all other factors), the overall QoL score changes from 38.23 to 43.33, that is, the QoL worsens. In this model, these predictors, combined, explain approximately 46% of the variations among the study participants in the overall QoL (*R* ^2^ = 0.455; Table 5).

**TABLE 5:** Model summary.

Model	*R*	*R* square	Adjusted *R* square	Std. error of the estimate
1	0.675	0.455	0.412	16.49641

## 4. Discussion

The main purpose of the current study was to examine the relationships between HD patients’ HRQoL and its possible determinants, including comorbidities, smoking status, climate change‐related factors, and place of residence. The results showed that education level, related illnesses, workplace environment, smoking, stress, and pain were significant independent predictors of the overall QoL, accounting for about 46% of the variance (*R* ^2^ = 0.455). These findings add to the scant literature on the variables affecting HRQoL in this susceptible group [7], especially when considering the Middle East and North Africa (MENA) region, where quick changes in the environment and way of life may have a special impact on patient outcomes [9]. Studies show that compared to the general population, HD patients are more likely to experience reduced QoL [40]. Gerasimoula et al. found that overall QoL scores were higher among those under 60 [41]. A study by Anees et al. [42] revealed that five variables—age, race, employment status, income, and duration of HD—correlated with a lower QoL for dialysis patients with chronic renal disease. Comorbidity had an effect on the HD patients’ QoL in this study. A higher number of comorbidities have had an impact on lower QoL. The results of this study are understandable because patients’ physical, emotional, and social conditions typically worsen as comorbidities increase [43]. Approximately 10% of American adults have osteoarthritis (OA), which has a negative impact on their QoL. OA reduces QoL in older people due to discomfort, functional limitations, and depression [44].

Significant correlations were found in the current study between patient QoL and a number of variables. A high QoL was associated with being younger, single, more educated, having a higher family income, and having better employment opportunities. Gender did not correlate with QoL in the interim. Clinical factors such as the absence of hypertension and diabetes prevented a decline in QoL. Physical symptoms such as cramping, muscle soreness, and shortness of breath had a major negative impact on QoL [45]. HD’s effect on HRQoL is a major concern raised by patients. Mharapara et al. [46] provided strong evidence that the HRQoL of women receiving HD for renal failure is said to be lower than that of men. The impact of biological sex and sociocultural gender characteristics on the effect of HD dose on HRQoL is ignored by the widely used “one size fits all” approach to HD therapy. Women’s higher dialysis clearance and higher survival rates than men’s could be explained by biological differences such as smaller bodies and different water distribution. As a result, gender disparities in clearance may have a negative impact on women’s HRQoL [46].

Better QoL was significantly correlated with comorbidities (*β* = 1.732, *p* = 0.04). This result seems to be at odds with most of the literature currently in publication, which generally states that HD patients’ QoL is lower when they have a higher burden of comorbidities, such as diabetes, cardiovascular disease, and hypertension [33, 47]. As mentioned in the Introduction, diabetes and hypertension are known to have adverse health effects on HRQoL due to the burden of medication therapy as well as direct physiological effects [7]. Regarding the patient’s place of residence, social interaction differs between rural and urban environments, according to both conventional and contemporary sociological theories. Rural areas are typically characterized by close‐knit, informal interactions (strong ties), whereas urban areas may have more formal, rationalized interactions (weak ties). The literature on aging and social support emphasizes the importance of social interaction as a predictor of older adults’ health. Visits from friends, neighbors, or family were found to have a greater positive impact on the subjective well‐being of older adults in rural areas than on their urban counterparts [48].

In the current study, education level was found to be a significant negative predictor of QoL (*β* = −5.456, *p* = 0.001), meaning that lower educational attainment was linked to lower QoL. This result is in line with earlier studies showing better QoL outcomes are reported by patients with higher levels of education [7, 8]. It was found that anxiety, depression, and psychological distress related to climate change were more prevalent in younger patients. Positive correlations between anxiety, mental distress, and depression were discovered in relation to climate change. Additionally, a highly significant difference was noted across demographic factors such as age and education level [35]. It is becoming more widely acknowledged that people’s QoL is significantly influenced by the psychological effects of climate change. Natural disasters, job losses, and future uncertainties can exacerbate stress, anxiety, depression, and other mental health problems. In the context of global warming, particularly vulnerable groups, such as children, older people, and people with pre‐existing mental health problems, are more likely to suffer negative mental health outcomes. A broadly similar point has also been made by Cianconi et al. [49], who pinpoint a positive correlation between psychological suffering and worries about global warming, particularly psychological distress indicators such as stress, anxiety, and depression. A significant negative predictor of QoL was found to be occupational heat exposure (*β* = −8.229, *p* = 0.04), with exposed patients reporting lower QoL than their nonexposed counterparts. This result is consistent with recent research showing that climate change considerably lowers QoL in the areas of wellness, community, and finances [26–28].

The findings identify a negative correlation between smoking and HRQoL. Those who never smoked had higher HRQoL scores and fewer problems in the EQ‐5D dimensions. Additionally, compared to light and moderate smokers, heavy smokers’ HRQoL was generally lower. The smokers’ HRQoL scores and nicotine consumption were correlated [29]. However, there is contradictory evidence [36], where they found that smoking was linked to improved physical HRQoL in younger HD patients, indicating that this relationship may change with age. This was supported by another evidence [50], which reveals that smoking had an appreciable effect on the QoL of dialysis patients. Given that smoking is a modifiable risk factor that could be addressed through behavioral and nutritional interventions [33], the conflicting results in the literature highlight the need for additional research to elucidate the complex relationship between smoking and HRQoL in this population [35, 37]. The patient’s well‐being was adversely affected by social and environmental factors, including family distress and financial hardships. Psychological factors such as anxiety, depression, and fear of dying also had a negative impact on the QoL. According to a different study [38], men’s QoL was solely influenced by their depression level, but women’s QoL was predicted by both pain intensity and depression level. This implies that the factors that determine QoL differ by gender. It was suggested that women might be more vulnerable than men to both physical and psychological stressors [45].

Relevant strategies can account for the underlying causes of the observed gender differences in QoL. Poorer QoL was linked to higher levels of stress (*β* = 0.895, *p* = 0.04). This result is in line with a substantial amount of research showing the negative effects of psychological stress on outcomes related to physical and mental health in populations with chronic illnesses [9, 34]. In addition to other psychosocial factors, stress may influence the nutritional status by altering appetite, reducing dietary adherence, and affecting metabolic control [37]. In the multivariate model, the strongest predictor of QoL (QoL) was pain (*β* = 5.111, *p* = 0.001), with lower QoL being linked to higher pain scores. This result is in excellent agreement with earlier research that repeatedly shows pain to be a significant factor in determining QoL in dialysis patients [7–9]. From a nutritional standpoint, chronic pain may make it more difficult for patients to manage their metabolism by interfering with food intake, hydration, and adherence to dietary recommendations [37]. Treatment for renal failure that is patient‐centered and holistic is becoming more widely acknowledged as a crucial addition to traditional care. These methods place a strong emphasis on interdisciplinary care teams, which include nephrologists, nurses, dietitians, psychologists, and social workers; coordinated care pathways; shared decision‐making; and consideration for patients’ values, objectives, and QoL [17, 51].

## 5. Conclusion

The results of the present study demonstrate that HD patients show impairments in the clinical, physical, social, and psychological facets of QoL. In effect, these results highlight the value of patient‐centered, holistic approaches to renal failure treatment. For these patients, tailored interventions that address their diverse needs can enhance their QoL and promote the best possible health outcomes. To build on these results and guide the development of targeted interventions aimed at promoting patient welfare and QoL in HD settings, further, especially longitudinal, research is necessary.

## Author Contributions

Amany Gomaa Atia and Huda Badr Abd Elhamed developed the initial concept of this study. Hanaa Elsayed Elsayed Mohamed and Shimaa Galal El Sayed collected the data and performed the analysis. Hani Jameel Hamad and Mohamed Ali Elsayed wrote the first draft of the manuscript. Amany Gomaa Atia and Mohamed Ali Elsayed performed the final editing of the manuscript.

## Funding

This research received no funding.

## Disclosure

All authors have read and agreed to the published version of the manuscript.

## Ethics Statement

Ethical approval was obtained from the Research Ethics Committee of Sinai University (Ref. No: SU.REC.2025 (46 H).

## Conflicts of Interest

The authors declare no conflicts of interest.

## Data Availability

The data that support the findings of this study are available from the corresponding author upon reasonable request.
